# The Expression of the StNRAMP2 Gene Determined the Accumulation of Cadmium in Different Tissues of Potato

**DOI:** 10.3390/ijms24119322

**Published:** 2023-05-26

**Authors:** Yule Zhang, Tengbing He, Weijun Tian, Yabei Xia, Yeqing He, Minmin Su, Guandi He

**Affiliations:** 1 College of Agriculture, Guizhou University, Guiyang 550025, China; gs.yanzhao21@gzu.edu.cn (Y.Z.); tbhe@gzu.edu.cn (T.H.);; 2 Institute of New Rural Development, Guizhou University, Guiyang 550025, China; 3 Research and Development Center of Fine Chemical Industry, Guizhou University, Guiyang 550025, China; gs.ybxia21@gzu.edu.cn

**Keywords:** transcriptome, functional verification, heterologous expression, VIGS

## Abstract

Cadmium (Cd) is a toxic metal that threatens human health when enriched in crops. *NRAMP*s are a family of natural macrophage proteins reported to play a key role in Cd transport in plants. In order to explore the gene regulation mechanism of potato under Cd stress and the role of *NRAMP*s family in it, this study analyzed the gene expression differences of two different Cd accumulation levels in potato after 7 days of 50 mg/kg Cd stress and screened out the key genes that may play a major role in the differential accumulation of Cd in different varieties. Additionally, *StNRAMP2* was selected for verification. Further verification showed that the *StNRAMP2* gene plays an important role in the accumulation of Cd in potato. Interestingly, silencing *StNRAMP2* increased Cd accumulation in tubers but significantly decreased Cd accumulation in other sites, suggesting a critical role of *StNRAMP2* in Cd uptake and transport in potatoes. To further confirm this conclusion, we performed heterologous expression experiments in which overexpression of *StNRAMP2* gene in tomato resulted in a threefold increase in Cd content, which further confirmed the important role of *StNRAMP2* in the process of Cd accumulation compared with wild-type plants. In addition, we found that the addition of Cd to the soil increased the activity of the plant antioxidant enzyme system, and silencing *StNRAMP2* partially reversed this effect. This suggests that the *StNRAMP2* gene plays an important role in plant stress tolerance, and future studies could further explore the role of this gene in other environmental stresses. In conclusion, the results of this study improve the understanding of the mechanism of Cd accumulation in potato and provide experimental basis for remediation of Cd pollution.

## 1. Introduction

Inappropriate human production activities can lead to excessive heavy metal residues in soil [1], For example, industrial discharges and improper mining of mineral veins will further exacerbate soil heavy metal pollution [2]. These toxic metals will spread to the human body through crops; damage bones, kidneys and other organs; and seriously affect human health [3,4,5]. Cadmium (Cd) is a group of carcinogens. Long-term exposure to Cd can lead to high blood pressure, decreased kidney function, and even an increased chance of cancer [6,7]. The accumulation of Cd by plants (especially crops) is a direct threat to human life and safety [8]. Cd-enriched plants can effectively separate and reduce Cd content in soil [9,10]. However, due to cost and technical reasons, it cannot be widely used in farmland in the short term. Therefore, the selection and seeding of low Cd accumulation crop varieties has become the main measure to reduce the intake of Cd in the human body [11].

Potatoes are the world’s fourth largest food crop after wheat, rice and maize, and are widely grown around the world [12], The growth and quality of potato tuber will be seriously affected by abiotic stress in natural environment [13]. In high Cd soil, potato tubers can accumulate excessive Cd [14], which leads to excessive Cd intake and affects the health of consumers [15]. Previous studies have shown that different varieties of potato have significant differences in the degree of Cd accumulation [16,17]. The laboratory conducted Cd accumulation screening on different varieties of potatoes in the early stage and screened out the cultivar Yun Shu 505 (YS505) with low Cd accumulation and the cultivar Wei Yu No.7 (WY7) with high Cd accumulation in tuber. Since Cd absorption is mainly passive transmembrane transport, the differences in Cd accumulation sites in different varieties of potatoes are likely to be directly affected by differences in functional gene expression.

Potato is a plant from the Nightshade family, which has been propagated asexually for a long time. YS505 and WY7 are homologous tetraploid varieties with highly heterozygous genomes, which affect and hinder the genetic analysis and improvement of potato [18]. The dwarf tomato variety (Micro-Tom) is a model Solanaceae plant, which is similar to Arabidopsis thaliana and has the characteristics of small size, short cycle, and ease of obtaining mutant genetic materials. It is widely used in the study of functional genes in Solanaceae [19,20]. VIGS (virus-induced gene silencing) has been widely used in the field of genetics to identify genes involved in various biological processes in many plant species [21,22]. Gene silencing can also be triggered in tetraploid potato, which is an effective way to observe the developmental mechanism in asexual propagation [23,24].

NRAMP (Natural resistance-associated macrophage protein), a natural macrophage protein associated with plant resistance, is involved in the transport of various metal elements in plants [25,26]. *BcNRAMP1* in Chinese cabbage can promote the absorption of Cd in Chinese cabbage leaves, and the mutation of *BcNRAMP1* in Arabidopsis thaliana can effectively reduce the accumulation of Cd [27]. In rice *OsNRAMP*s, *OsNRAMP1* and *OsNRAMP5* are significantly expressed under Cd stress [28]. *FeNRAMP5* in buckwheat showed transport activity for Cd and manganese, and excessive expression could promote the uptake of manganese in the root system [29]. At the same time, the expression of *MhNRAMP1* was significantly upregulated under the background of Cd and calcium in Calla Lily [30]. *NRAMP2* is localized in the trans-Golgi network, and overexpression can effectively improve plant growth under low manganese conditions [31]. These results indicate that *NRAMP*s plays a significant role in plants’ absorption of heavy metals.

In this study, potato varieties with low Cd accumulation (YS505) and high Cd accumulation (WY7) were selected and cultivated under 50 mg/kg Cd stress. Transcriptome analysis was used to analyze the gene expression differences between 0 d and 7 d stress, and the candidate genes related to heavy metals with significant differences were screened out to explore the role of *NRAMPs* gene family in the change of Cd concentration in potato tubers. In this study, *StNRAMP2* was silenced in potato and heterologously expressed in tomato. In this study, we focused on the effects of *StNRAMP2* on plant growth and its role in Cd accumulation in the context of Cd and analyzed its effects on the REDOX levels of potato. The aim is to provide a new idea for the breeding of potato varieties with low Cd accumulation.

## 2. Results

### 2.1. Gene Expression Patterns of Potato under Cadmium Stress for Different Days

Transcriptome analysis was performed using the potato varieties YS505 and WY7 after 7 days of stress in 50 mg/kg cadmium background soil (BioProject: PRJNA961841). The original data obtained via sequencing and the data after quality control were statistically analyzed. The percentage of Q20 is greater than or equal to 97.7%, the percentage of Q30 is greater than or equal to 93.25%, and the percentage of GC ranges from 42.9% to 43.37% (Tables S1 and S2, Figure S1).

**Figure 1:**
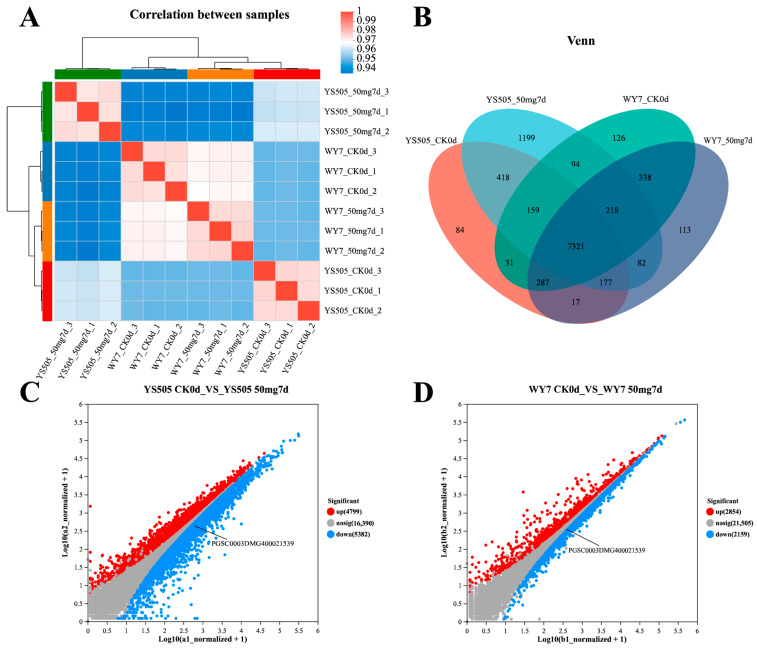
Analysis of gene expression between transcriptome samples: (**A**) shows the correlation among samples; (**B**) is a Venn diagram, in which circles of different colors represent a processed sample, genes screened based on expression level >10, and values represent the number of common and unique genes among samples of different treatments. The sum of all numbers inside the circle represents the sum of the number of genes in the processed sample, and the cross areas of the circle represent the total number of genes in each sample. (**C**,**D**) show the gene expression differences before and after cadmium stress of YS505 and WY7, respectively. The horizontal and vertical coordinates represent the gene expression levels in the two samples, respectively, with each dot representing a specific gene.

The sample correlation heat map analysis showed that there were significant differences in YS505 samples before and after treatment (Figure 1A), and the overall gene expression of YS505 was significantly upregulated after cadmium stress (Figure 1B). There were 4799 upregulated genes and 5382 downregulated genes in YS505. There were 2854 upregulated genes and 2159 downregulated genes in the variety WY7 (Figure 1C). A Venn diagram was used to display statistics on genes with expression levels > 10 in different samples, and 1199 genes were significantly upregulated under YS505 cadmium stress within a period of 7 days (Figure 1D). This was the sample with the most significant gene expression differences.

### 2.2. Identification of Metal Tolerance Genes

**Figure 2:**
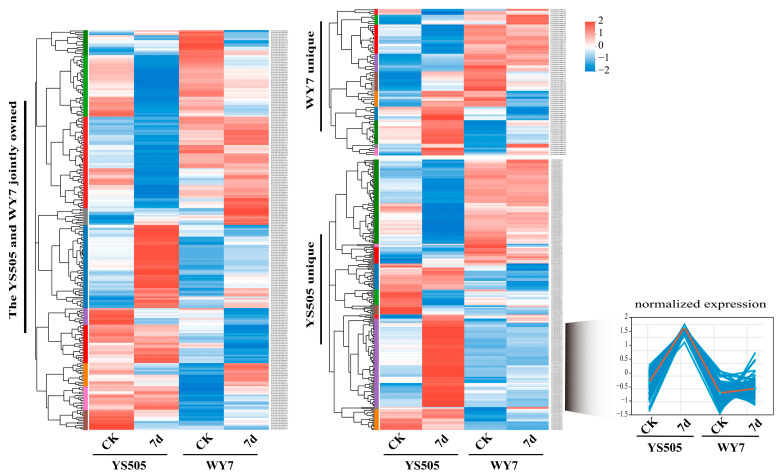
Map of gene expression differences among potato varieties: Each row represents a gene in the heat map of gene clustering, and the color represents the level of expression (TPM-log10). The specific variation trend of the expression level is marked on the upper right, the tree diagram of gene clustering and the module diagram of subclustering are on the left, and the name of the gene is on the right. The closer two gene branches are, the closer their expression levels are.

In order to analyze the difference of gene expression levels of YS505 and WY7 under Cd stress for 7 days, 575 high-expression genes related to medium heavy metal were selected from the two varieties under the condition of (*p* < 0.01, expression level > 10), among which 260 were specific to YS505, 75 were specific to WY7, and 240 were shared between the two varieties. In order to identify their functions, cluster heat maps were used to perform sub-cluster analysis on the specific genes and common genes of the two varieties, respectively (Figure 2A). In order to investigate to what extent gene expression in potato leaves can promote cadmium accumulation in leaves and reduce cadmium content in tuber, 85 genes with high and significant expression were selected to construct gene sets specific to YS505, a cultivar with low Cd accumulation in tuber, after stress. This included *StYSL1* (PGSC0003DMG400012242), *StATP23* (PGSC0003DMG400011703), *StHIPP35* (PGSC0003DMG400024753), *StHIPP36* (PGSC0003DMG400011344), *StNAKR2* (PGSC0003DMG400017602), and *StNRAMP2* (PGSC0003DMG400021539), etc. (Table S3), which are often reported to play a key role in Cd absorption.

**Figure 3:**
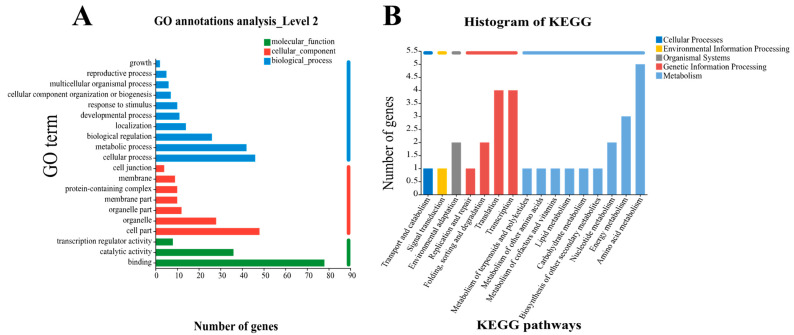
Mapping and classification of Cd-related genes. (**A**) is a bar chart of GO classification statistics (single gene set): In the figure, the vertical axis represents the second-level classification terms of GO, the horizontal axis represents the number of genes compared to the second-level classification, and the three colors represent the three classifications. (**B**) is the enrichment diagram of the KEGG signal pathway. Ordinate is the name of the KEGG metabolic pathway. The x-coordinate is the number of genes or transcripts annotated to this pathway. The upper right corner shows the category of KEGG metabolic pathways.

For a more intuitive understanding of differentially expressed genes, the GO classification statistical bar chart was drawn (Figure 3A), in which Biological Process and BP recorded the most GO terms, followed by Cellular Component, CC, Molecular Function, and MF. The Cellular Component (CC) is more involved in the cell part (GO:0044464); Genes in Biological Process (BP) are more involved in cellular process (GO:0009987). Molecular Function (MF) is mainly involved in binding (metal ion binding, protein binding, ATP binding, etc.). KEGG was used to analyze its metabolic pathway (Figure 3B), and the number of genes involved in energy metabolism was the largest (Figures S2 and S3, Table S4). In order to analyze the role of the *NRAMPs* family in Cd transport in potato, we focused on *StNRAMP2*, which has significantly different expression levels in different samples and performs metal transport functions, for verification (Figure S4).

### 2.3. Qualitative and Quantitative Analysis of StNRAMP2

**Figure 4:**
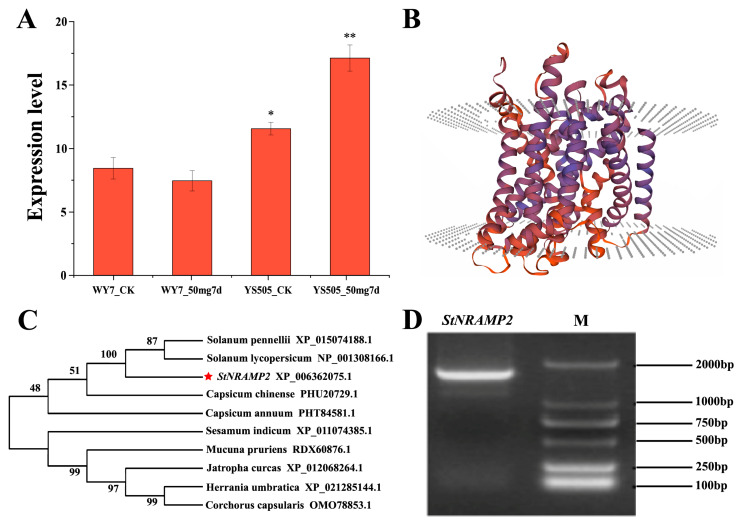
Qualitative and quantitative study of *StNRAMP2*: (**A**) shows the expression of *StNRAMP2* in different samples. In the figure, * means significant difference *p* < 0.05, and ** means extremely significant *p* < 0.01. (**B**) is the three-dimensional structure diagram of *StNRAMP2*. (**C**) shows the evolutionary tree drawn by *NRAMP*s members of different species with high similarity to *StNRAMP2*. (**D**) shows the PCR electrophoresis of *StNRAMP2* gene cloning.

*StNRAMP2*, which is significantly expressed in potato Cd uptake by *NRAMPs* family, was screened out from 85 stably upregulated genes for expression analysis among different samples, and its expression was significantly upregulated in YS505 (Figure 4A). Amino acid sequence analysis showed (Figure S1) that the coding region of *StNRAMP2* gene consisted of 1623 base pairs encoding 540 amino acids, with a theoretical isoelectric point of 5.00 and a predicted relative molecular weight of 134.95 kDa. *StNRAMP2* gene encodes A variety of amino acids: T (Threonine) (31.7%), G (Glycine) (26.6%) and Alanine (22.1%). The three-dimensional structure diagram is shown in (Figure 4B and Figure S5).

Blast comparison of potato StNRAMP2 sequences in NCBI was conducted. Solanum pennellii, Solanum lycopersicum, Capsicum chinense, Capsicum annuum, Sesamum indicum, Herrania umbratica, Jatropha curcas, Corchorus capsularis, Mucuna pruriens and other species with high similarity were selected to construct the phylogenetic tree (Figure 4C). The results showed that StNRAMP2 protein is closely related to Solanum lycopersicum and Solanum pennellii in adjacent branches but is only distantly related to Corchorus capsularis and Herrania umbratical (Table S5).

According to the CDS sequence of potato *StNRAMP2* gene that was identified, YS505cDNA was used as a template for PCR amplification using *StNRAMP2* primer, and a single band of about 1800 bp was obtained via electrophoresis detection (Figure 4D). The target band was recovered, connected to the cloning vector, transformed into *Escherichia coli,* and screened for positive clonal colonies. The sequencing was sent to Shenggong Bioengineering (Shanghai, China) Co., LTD., and the sequencing results were basically consistent with CDS sequences in the NCBI database (Table S6).

### 2.4. Cloning and Expression of StNRAMP2

**Figure 5:**
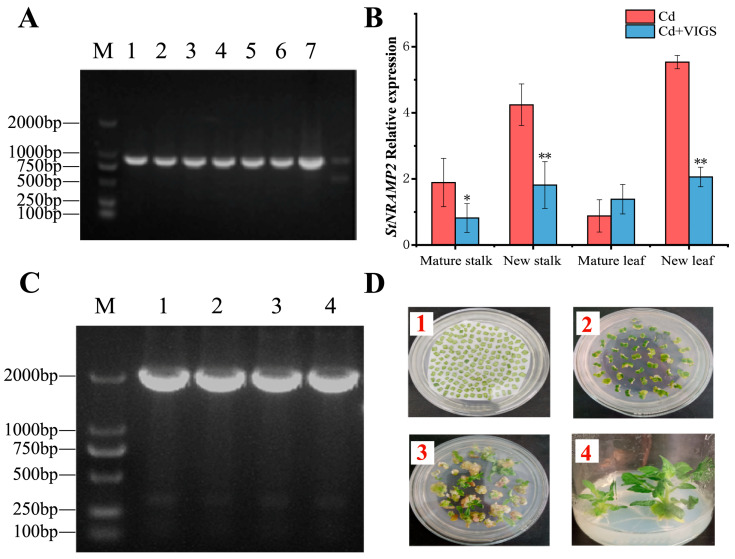
Cloning and transformation of *StNRAMP2*: (**A**) shows the PCR results of Agrobacterium Tumefaciens colonies (M: DL2000; 1–6: pTRV2 + *StNRAMP2* gene; 7: plasmid; 8: water); (**B**) shows the expression of TRV-*StNRAMP2* gene silenced plants. In the figure, * means significant difference *p* < 0.05, and ** means extremely significant *p* < 0.01. (**C**) shows the colony PCR detection of the part-CAM-FLAG-*StNRAMP2* plasmid, M is the DL2000 marker, and 1–4 are the colony PCR results of the target gene. (**D**) Genetic transformation process of part-CAM-FLAG-*StNRAMP2* tomato, 1: Preparation of explants; 2: callus formation; 3: resistant bud formation; 4: rooting culture.

According to the *StNRAMP2* gene sequence, a 362 bp target sequence was designed and synthesized (Figure S6), and a single colony was selected for PCR detection of the target gene colony (Figure 5A). The results showed that the plasmid pTRV2 + *StNRAMP2* was successfully transferred into Agrobacterium GV3101. The mature potato leaves were injected into the main vein of the back of the leaves with a disposable sterile medical pinhole syringe for *StNRAMP2* silencing, and the expression level of the silenced plants was measured. The relative expression of *StNRAMP2* gene (Figure 5B) showed that the overall expression of *StNRAMP2* gene in potato plants was relatively low, and the silencing effect was good. Compared with plants without silencing treatment, the relative expression of *StNRAMP2* gene in young stems and new leaves significantly decreased: by 2.3 times and 2.7 times, respectively. The relative expression in mature stems was significantly lower than CK. In old leaves, gene silencing did not significantly reduce the expression of *StNRAMP2* gene but increased the expression.

The plant expression vector of CaMv35S promoter (Figure S7) was constructed using a homologous recombinant cloning technique and transformed into *Escherichia coli* receptive cells. Colony PCR verification was performed, and the colonies were identified as positive recombinant transformants to extract the plasmid (Figure 5C). The recombinant plasmid was transformed into Agrobacterium Tumefaciens (GV3101), and the tomato genetic transformation of *StNRAMP2* was carried out using an Agrobacterium-mediated method (Figure 5D and Figure S8).

### 2.5. Silencing StNRAMP2 Significantly Affected Potato REDOX Levels

**Figure 6:**
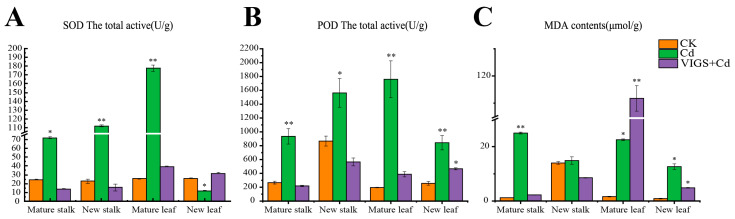
Effect of *StNRAMP2* silencing on the REDOX level of potato plants: (**A**) shows SOD activity in different tissues of potato under Cd stress. (**B**) shows POD activity in different tissues of potato under stress. (**C**) shows MDA content in different tissues of potato after stress. In the figure, * means significant difference *p* < 0.05, and ** means extremely significant *p* < 0.01.

The effect of Cd stress on the activity of superoxide dismutase (SOD) in potato tissues is shown in Figure 6A. Compared with CK plants, Cd treatment significantly increased SOD activity in mature stems, young stems, and old leaves of potato and significantly decreased SOD activity in *StNRAMP2* silenced plants, but significantly increased SOD activity in young leaves. In mature leaves, the SOD activity of Cd treatment was the highest at 177.47 U/g, and SOD content of VIGS + Cd treatment was 39.53 U/g, which was reduced by 77.7% compared with Cd treatment, followed by 112.04 U/g in young stems and 15.78 U/g in VIGS + Cd treatment. Compared with Cd treatment, there was a reduction of 85.9%.

The effect of Cd stress on the activity of peroxidase (POD) in potato tissues is shown in Figure 6B. In mature stems, young stems, old leaves and new leaves, POD content showed a trend of first increasing and then decreasing under different treatments, and the content was CK < Cd > VIGS + Cd. *StNRAMP2* silencing could significantly reduce POD content in each part of potato. Especially in mature leaves, the content of Cd treatment was significantly different at 1760.00 U/g, whereas VIGS + Cd treatment was 387.75 U/g, which was 77.99% lower than that of Cd treatment.

The effect of Cd stress on malondialdehyde (MDA) content in potato tissues is shown in Figure 6C. *StNRAMP2* silencing significantly decreased MDA content in mature stems, young stems and new leaves, and significantly increased MDA content in VIGS + Cd in mature leaves. *StNRAMP2* silencing significantly reduced the overall oxidation activity level of potato, especially in stems.

### 2.6. StNRAMP2 Was Positively Correlated with Cadmium Tolerance in Plants

**Figure 7:**
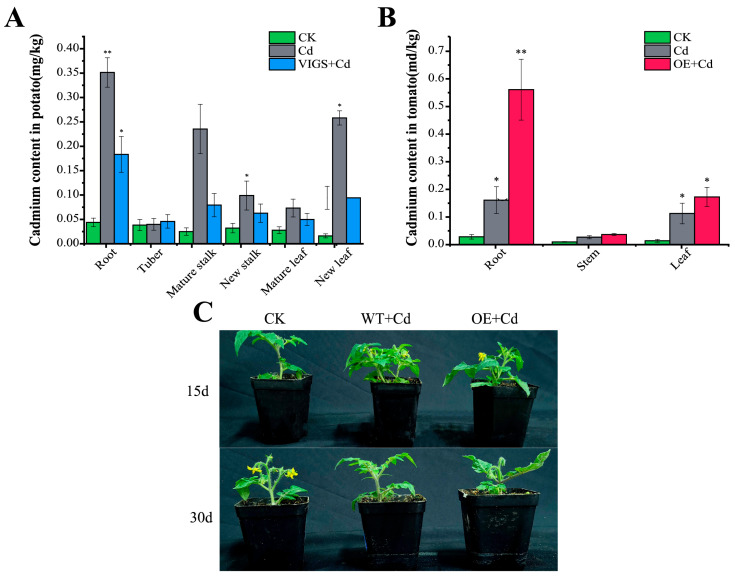
Effect of *StNRAMP2* on Cd tolerance in plants: (**A**) shows Cd accumulation in different parts of potato. (**B**) shows Cd accumulation in different parts of tomato. In the figure, * means significant difference *p* < 0.05, and ** means extremely significant *p* < 0.01. (**C**) shows tomato under heavy metal stress. CK was a plant without Cd stress. WT is a wild-type plant; VIGS signifies silenced *StNRAMP2* plants; OE is a plant that overexpresses *StNRAMP2*.

CK is the plant without Cd stress; Cd is the plant under Cd stress only; VIGS + Cd was used for *StNRAMP2* silenced plants under Cd stress; OE + Cd was the *StNRAMP2* overexpressing plant under Cd stress. One month later, the heavy metal content in tissues of potato plants after various treatments was determined (Figure 7A). The results showed that Cd mainly accumulated in potato roots, followed by mature tissues and organs. The Cd content in CK-treated plants was 0.016 ± 0.004~0.044 ± 0.009 mg/kg, the Cd content in Cd-treated plants was 0.040 ± 0.012~0.351 ± 0.030 mg/kg, and the Cd content was root > old leaf > mature stem > young stem > new leaf > tuber. The Cd content in VIGS + Cd treatment ranged from 0.046 ± 0.014 to 0.183 ± 0.037 mg/kg, and the Cd content was in root > old leaves > mature stems > young stems > new leaves > tubers. After *StNRAMP2* gene silencing, the Cd content in other tissues decreased, except in tubers, and the decrease was the highest in mature stems. It decreased by 0.156 mg/kg and 66.30% compared with unsilenced plants. Secondly, in the old leaves, Cd concentration was reduced by 0.164 mg/kg (63.48%) compared with the treatment without gene silencing, and Cd content in the leaves was reduced. Silencing *StNRAMP2* increased Cd accumulation in tuber, the main edible part. These results indicated that *StNRAMP2* silencing could increase the accumulation of Cd in potato tuber and decrease the content of Cd in other parts. *StNRAMP2* played a role in promoting the accumulation of Cd in potato roots, stems, and leaves, and inhibited the accumulation of Cd in potato tuber.

In order to verify the absorption and transport effect of *StNRAMP2* on Cd again, this gene was over-expressed in tomato, and samples were collected and Cd content was measured at 30 days. The results are shown in Figure 7B. After 30 days of Cd stress, the Cd content was CK < Cd < OE + Cd, and the difference was the most significant in roots. At 30 days of Cd stress, the Cd content of OE + Cd plants increased by three times compared with that of Cd plants. In leaf tissue, it increased by 1.6 times. These results indicated that *StNRAMP2* could significantly increase Cd accumulation in plant roots and leaves. According to the growth appearance and morphology of tomatoes, the plant biomass was higher at 15 days of Cd stress than at 30 days of Cd stress, and the growth state was better. After 30 days of Cd stress, plant leaves were smaller, flowers fell off, and plant height did not change significantly (Figure 7C).

## 3. Discussion

### 3.1. RNA-Seq Screened 85 Cadmium Tolerance Genes in Potato

Through transcriptome analysis, researchers discovered that the expression of potato genes varies greatly among different varieties [32], which directly affects individual differences in potato growth. Among cultivars, there are significant variations in the expression profiles of genes that affect tuber development [33]. The variation in gene expression is more prominent in potatoes with high heterozygosity and tetraploid inbreeding inhibition [34]. In order to understand the key genes that lead to the differences in Cd absorption levels among different varieties, 575 genes with high expression related to heavy metals were selected by analyzing the gene expression differences of YS505 and WY7 under Cd stress, among which 260 were specific to YS505, 75 were specific to WY7, and 240 were shared between the two varieties. A total of 85 functional genes were identified from the Cd-tolerant YS505 by subcluster analysis. This will provide a good basis for exploring the reasons for the differences in Cd accumulation among potato varieties. GO analysis revealed that Biological Process (BP) had the largest number of GO terms, whereas the Cellular Component (CC) was predominant in the cell part (GO:0044464). Genes in BP were mainly associated with the cellular process (GO:0009987), whereas Molecular Function (MF) was primarily related to binding (metal ion binding, protein binding, ATP binding, etc.). KEGG analysis of metabolic pathways showed that the energy metabolism pathway involved the largest number of genes.

### 3.2. The Expression of StNRAMP2 Was Positively Correlated with the Accumulation of Cadmium in SOLANACEAE

The *NRAMP*s gene family has been extensively studied for its role in facilitating the transportation of heavy metals such as Cd, Fe, and Mn in a variety of plant species [35,36], Fe transport has been disputed in some studies, and the barley (Hordeum vulgare) *HvNRAMP5* protein mediates the absorption of Cd and manganese but not iron [37]. However, the effect of *NRAMP* protein on Cd absorption in potatoes remains unexplored. In order to gain a deeper understanding of the role of *NRAMP* protein in Cd accumulation within potato tubers, we endeavored to clone *StNRAMP2* from the potato *NRAMP* gene family. Through the utilization of virus-induced gene silencing (VIGS) methodology, we silenced the expression of *StNRAMP2* in potatoes and obtained transgenic plants in which *StNRAMP2* was overexpressed in tomato Solanaceae. By measuring the expression level of the *StNRAMP2* gene and comparing the Cd content in silent and normal plants, we investigated the effect of the *StNRAMP2* gene on Cd transport and absorption in potatoes.

In rice, research has shown that overexpression of *OsNRAMP5* reduced the transport of Cd from roots to buds and resulted in a decrease in Cd accumulation in rice grains. Conversely, silencing *OsNRAMP5* can improve Cd tolerance in rice, thereby reducing overall Cd accumulation [38,39]. When comparing Cd treatments in potato tissue, it was found that Cd was mainly concentrated in the roots. The content of Cd in potato tissues treated with Cd was found to be highest in the roots, followed by the stem and then tuber. In *StNRAMP2*-silenced plants, there was a slight increase in Cd content in tubers but a decrease in other tissues. This could suggest that silencing *StNRAMP2* reduces the rate of Cd migration from tubers to leaves. It should be noted that overexpression of *StNRAMP2* can reduce Cd tolerance in tomatoes, as Cd content in rhizomes and leaves increases, indicating that *StNRAMP2* plays a role in Cd migration in plants. These findings provide valuable insight for regulating Cd accumulation in different plant tissues.

### 3.3. Silencing StNRAMP2 Decreased the Activity of Antioxidant Enzymes in Different Tissues of Potato

Heavy metal stress can have various degrees of toxicity for plants and affect their physiological processes, leading to an increase in the concentration of reactive oxygen species (ROS) [40]. ROS are metabolic substances formed during plant metabolism by electron transfer in chloroplasts, mitochondria, and plasma membrane. Although production and elimination of ROS are balanced, they can accumulate rapidly owing to different abiotic stresses [41]. Such accumulation causes a disruption in the balance between ROS production and antioxidants systems, ultimately leading to damage to the cell membrane, nucleic acid, and chloroplast pigment [42], Malondialdehyde (MDA) is the final product of lipid peroxidation in plant cell membranes caused by abiotic stress and is the main standard by which to measure oxidative damage (lipid peroxidation level) [43,44]. To combat the damage of ROS accumulation and protect macromolecules from oxidative damage, plants have accordingly developed a powerful antioxidant system consisting of catalase (CAT), superoxide dismutase (SOD), peroxidase (POD), ascorbate peroxidase (APX), etc. [45,46].

This study found that plants treated with Cd had higher heavy metal concentrations than the control group, implying that the plants were subject to increased toxicity. The contents of POD, SOD, and MDA also increased, indicating that antioxidant capacity was enhanced. The contents of POD, SOD, and MDA were relatively low in young tissues such as new leaves and young stems, as was the concentration of Cd, which indicates that the stress effect was weak in the young tissues, since heavy metals were mainly enriched in older tissues. Silencing *StNRAMP2* resulted in decreased oxidation indexes and reduced damage to plants. *StNRAMP2* silencing significantly reduced REDOX activity in all tissues under Cd stress, reducing damage to all plant tissues. Consequently, Cd stress in potatoes was reduced after silencing of *StNRAMP2*.

## 4. Materials and Methods

### 4.1. Material Acquisition and Processing

#### 4.1.1. Carriers and Bacteriocins

Virus-induced gene silencing, VIGS (Ptrv1, Ptrv2); Silent carrier; part-CAM-FLAG expression vector; DH5α receptor cells; Agrobacterium agrobacterium (GV3101); pTRV and part-CAM-FLAG hyperexpression vectors were provided by Shaanxi Bo Rui De Biotechnology Co., LTD. (Xi’an, China).

#### 4.1.2. Plant Materials and Treatment

Plant materials: Potato (Yun Shu 505, YS505), (Wei Yu No.7, WY7), tomato (Micro-Tom); (Both were obtained from College of Agriculture, Guizhou University).

Sterile tomato material acquisition: Ripe tomato seeds were soaked in sterile water for 30 min. They were disinfected with 75% alcohol for 30 s, then disinfected with 2% sodium hypochlorite solution for 16 min, washed five times with sterile water, blotted dry with sterile water, and inoculated on 1/2 MS medium for light culture. After 8 days, the leaves of germfree seedlings were removed, both ends were removed, and the leaves were cut into a 0.5 cm square. The leaves were then cultured in the medium (MS + KT 1 mg/L pH 5.8) for 24 h.

Treatment: (1) Soil treatment: the yellow soil without cadmium pollution was air-dried and passed through a 4 mm sieve. After sieving, CdCl_2_ was added to the soil, and 81.5 mg CdCl_2_ was added per kilogram of soil. After mixing, the background soil containing Cd (50 mg/kg) was formed. Next, 163 mg CdCl_2_ per kg of soil was added, and the background soil containing Cd (100 mg/kg) was mixed. (2) Cadmium tolerance gene screening test: in order to excavate the related genes affecting cadmium accumulation in potato, the potato tubers of YS505 with low cadmium accumulation in tubers and WY7 with high cadmium accumulation in tubers were selected and planted in plastic POTS with the same background value of soil, germinated and placed outside for one month. Six potato plants with similar growth conditions were selected from the two varieties and transplanted into soil containing Cd (50 mg/kg), and their leaves were collected at 0 d and 7 d after transplanting. (3) RTqPCR verification experiment and oxidase activity experiment: potato plants silenced by *StNRAMP2* were grown in CdCl_2_ (100 mg/kg) supplemented soil for 30 days before sampling, and new leaves, mature leaves, new stems and mature stems were cut, respectively. (4) Cadmium-specific tissue accumulation experiment: Transgenic potato and tomato plants were transplanted into CdCl_2_ (100 mg/kg) soil under stress, and the difference of cadmium content in different tissues was determined after 30 days of growth.

### 4.2. Transcriptome Screening of Cadmium Tolerance Candidate Genes in Potato

#### 4.2.1. Transcriptome Sequencing

Two potato varieties were used to collect leaf tissues under cadmium stress at 0 d and 7 d, respectively. Total RNA was extracted from the tissue samples and its concentration and purity were evaluated using Nanodrop2000. The integrity of the RNA was assessed using agarose gel electrophoresis, while the RIN value was determined using Agilent2100. For a successful library construction, the total amount of RNA required should be at least 1 ug, with a concentration of at least 35 ng/μL, an OD260/280 ratio of ≥1.8, and an OD260/230 ratio of ≥1.0. In this study, mRNA samples from pepper plants before and after cadmium stress were sequenced using the Illumina Hiseq X ten (Illumina, San Diego, CA, USA) sequencing platform. Library construction was performed using the Illumina TruseqTM RNAsample prep Kit, and sequencing was conducted through https://cloud.majorbio.com (access date: 29 June 2022) [47].

To evaluate the quality of the sequencing data for each sample, statistical methods were employed, including base GC content, base mass distribution, and base error rate distribution statistics. The fastp tool (https://github.com/OpenGene/fastp; access date: 13 September 2022) was utilized for data quality control to obtain high-quality sequencing data, also known as clean data, to ensure smooth progress in subsequent analyses [48]. After performing quality control on the raw data to obtain clean reads, HiSat2 (http://ccb.jhu.edu/software/hisat2/index.shtml; access date: 16 September 2022) was used to map these reads against the reference genome. StringTie (https://ccb.jhu.edu/software/stringtie/; access date: 16 September 2022) was then employed to assemble the mapped reads and quantify transcript expression levels [49]. To evaluate the quality of the transcriptome sequencing comparison results, various metrics were considered, including sequencing saturation, gene coverage, and distribution of reads across different regions of the reference genome and chromosomes. The RSEM tool (https://deweylab.github.io/; access date: 9 October 2022) was utilized to calculate the quantity of gene expression [50] to facilitate subsequent analysis of gene differential expression among samples. DESeq2 was then used to screen for statistical significance in differences (DR < 0.01 (DESeq2), |log2FC| ≥ 1) to identify genes that exhibited significant changes in expression levels between the samples [51].

#### 4.2.2. Transcriptome Analysis of Candidate Genes

In this study, cluster analysis was first performed on the expression levels of selected genes among samples [52,53]. Again, through the GO (Gene Ontology, http://www.geneontology.org/ (accessed on 21 September 2022)) analysis was then used to classify genes based on their participation in biological processes (BP), cellular components (CC), and molecular functions (MF). To obtain a more direct understanding of the functional enrichment of differentially expressed genes, GO functional significant enrichment analysis was conducted for these genes to explain the differences among samples at the level of gene function [54]. The KEGG database (Kyoto Encyclopedia of Genes and Genomes, http://www.genome.jp/kegg/; access date: 16 September 2022) was used to classify genes according to their involvement in pathway signaling or functional pathways, with differentially expressed genes annotated accordingly. Finally, GSEA Version 3.0 (http://software.broadinstitute.org/gsea/index.jsp; access date: 9 October 2022) was employed for enrichment analysis.

### 4.3. Cloning and Transformation of StNRAMP2

The full-length coding frame sequence of the potato *NRAMP2* gene was obtained through a search of the National Center for Biotechnology Information (NCBI) database. Specific primers for *StNRAMP2* (F: TTTGGAGAGGACACGCTCGAGatgagctcccctccacagcaa, R: GTCATCCTTGTAGTCGAATTCttgaccagcatagctatatccttt) were designed based on this sequence, and the *StNRAMP2* gene was cloned from the low cadmium accumulation potato variety YS505.

Transient transformation of potato was achieved by silencing *StNRAMP2*. The pTRV2 + *StNRAMP2* plasmid was synthesized using the target sequence and transformed into Agrobacterium. The dry powder of the plasmid was dissolved in 30 μL sterilized water and verified after transformation. The primer sequence (TRVII-F: GAGTCCCACATATTCGCACG; TRVII-R: CCCCCCAACAATCTCTTAGC) was then detected. The pTRV1 and pTRV2 empty vectors were selected and cultured in YEP liquid medium containing 50 mg/L rifampicin (Kana) overnight at 28 °C with agitation at 200 RPM (r/min). The bacterial solution was then centrifuged at 4000 r/min for 10 min at 4 °C, resuspended in infection solution (10 mmol/L 2-molethoethonic acid +40 mg/L *Acetyleugenol* + 10 mmol/L magnesium chloride, pH5.6), and the concentration of the bacterial solution was adjusted to about OD600 = 1.0.

Next, pTRV1 was mixed with pTRV2 empty carrier and pTRV2-*StNRAMP2* in a 1:1 volume ratio, respectively, and left to stand for 4 h at room temperature in the dark. Mature potato leaves were selected and injected into the dorsal main leaf vein using a disposable sterile medical pinhole syringe. The leaves were incubated in the dark at 25 °C for 1 day and then transferred to normal light for 30 days, with normal water and fertilizer management. The plants were then colonized in 100 mg/kg cadmium soil.

Tomato Genetic Transformation with *StNRAMP2*: Explants showing healthy growth were mixed with a bacterial solution containing the plant expression vector for part-CAM-FLAG-*StNRAMP2*. The mixture was gently agitated for five minutes, and excess bacteria on the explant surface were removed using sterile filter paper. The explants were then cultured in the dark for two days and subsequently transferred to bud differentiation induction medium, with regular changes every other week. Once resistant buds had reached 2 cm in length, they were excised and transferred to rooting medium before being transplanted once roots had developed.

### 4.4. Expression Patterns of StNRAMP2 in Different Tissues under Cadmium Stress

The potatoes were silenced by *NRAMP2* and then subjected to cadmium stress at a concentration of 100 mg/kg. After one month, the plants were sampled and the new leaves, mature leaves, new stems, and mature stems were collected separately. RNA was extracted using the Easy Pure Plant RNA Extraction Kit (OMEGA, Guangzhou, China) and reverse-transcribed into cDNA with 3 biological replicates for each sample. Primers were synthesized by Shenggong Bioengineering (Shanghai) Co., LTD. (Shanghai, China). The Talent qPCR PreMix (Tiangen Biochemical Technology Co., LTD., Beijing, China) kit was used for real-time fluorescence quantitative PCR on a dedicated instrument. The data were calculated using the2^−ΔΔCt^ algorithm for analysis.

### 4.5. Determination of Antioxidant Activity of Potato

After one month of growth of wild-type potato strains and *StNRAMP2* silenced potato strains under cadmium stress, different growth sites were sampled, and biological repeats were performed three times for each growth site. Superoxide Dismutase, SOD, Peroxidase, POD and Malondialdehyde, MDA activities/contents were measured and compared with those of non-stressed potato strains to analyze the effects of *StNRAMP2* on cadmium tolerance of potato.

To measure the activity of SOD, the instructions of Solarbio (Beijing, China) superoxide dismutase activity detection kit (BC0175) were followed. For the detection and calculation of POD activity, the instructions of Solarbio (Beijing, China) peroxidase activity detection kit (BC0095) were referred to. To detect and calculate the content of MDA, the instruction manual of Solarbio (Beijing, China) Malondialdehyde (MDA) content Detection kit (BC0025) was consulted.

### 4.6. Determination of Cadmium Content in Plants

After a 30-day stress period, the cadmium content in plant tissues was determined using the GB/T5009 method. A dried sample weighing approximately 0.2000 g was dissolved on an electric plate using an HNO3-HClO4 (4:1) acid mixing system. Once cooled, the sample was filtered through a 0.45 µm filter membrane and diluted with ultra-pure water to 25 mL. The cadmium concentration was analyzed by an inductively coupled plasma emission spectrometer (iCAP 7000 ICP-OES, Thermo Fisher, Shanghai, China).

### 4.7. Statistic Analysis

The data were analyzed in Excel 2016, and the error line represents the standard error (SE). IBM spss Statistics 25 software was used. According to the *t*-test method, the significance level was * *p* < 0.05, ** *p* < 0.01. The bar chart is drawn using origin.

## 5. Conclusions

The following are the main conclusions of the study conducted: (1) Through the study of potato transcriptome, it was found that 85 genes in YS505 were significantly different and highly expressed under cadmium stress. (2) Silencing *StNRAMP2* has been found to increase cadmium content in potato tubers but significantly decreases it in other potato tissues. (3) Overexpression of *StNRAMP2* in tomato can reduce cadmium tolerance and increase the cadmium content in tomato plants, indicating that the *StNRAMP2* gene has a cadmium accumulation effect. (4) *StNRAMP2* silencing negatively affects the activity of the potato antioxidant enzyme system, which is otherwise activated due to the addition of cadmium. In summary, the study suggests that *StNRAMP2* may play a crucial role in reducing cadmium enrichment specifically in potato tubers by increasing the accumulation of cadmium in other parts of the potato and consequently reducing the content of cadmium in the main edible parts of the potato.

## Data Availability

Not applicable.

## Supplementary Materials

The following supporting information can be downloaded at: https://www.mdpi.com/article/10.3390/ijms24119322/s1.
