# Supplementary material for: The Expression of the StNRAMP2 Gene Determined the Accumulation of Cadmium in Different Tissues of Potato
Source: Int J Mol Sci. 2023 May 26;24(11):9322. doi: 10.3390/ijms24119322 (PMC10253094; doi:10.3390/ijms24119322)
Supplement: Supplementary file 1 [file ijms-24-09322-s001.zip › Supplementary Figures S1-S8.pdf]

Supplementary Materials

The expression of the StNRAMP2 gene determined the accumulation of cadmium in different tissues of potato

Supplementary Figures

Figure S1. Transcriptome sample expression and analysis.

Figure S2. Functional enrichment analysis of cadmium-related genes

Figure S3. GO enriched chorography.

Figure S4. Amino acid sequence analysis of potato StNRAMP2 gene

Figure S5. Qualitative StNRAMP2

Figure S6. Sequencing results of StNRAMP2 gene silencing fragment in potato.

Figure S7. Three-dimensional structure and protein interaction prediction.

Figure S8. PCR results of *StNRAMP2* transfection to tomato.

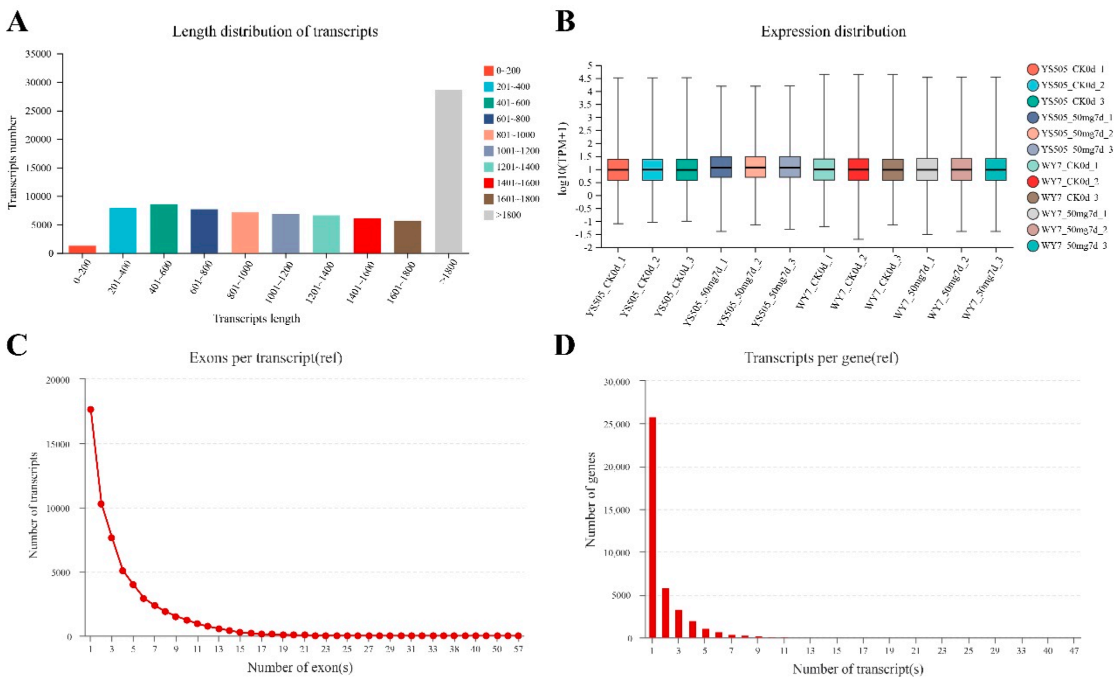

Figure S1. Transcriptome sample expression and analysis; Figure S1A shows the length distribution of transcripts. A bar chart shows the length distribution of different transcripts. The horizontal coordinate is the length range of transcript; The ordinate is the number of transcripts within the length range of the transcript; FIG. S1B is a box-shaped graph of expression quantity distribution: the abscis is the sample name, and the ordinate is the value of expression quantity after log10 logarithmic processing. Each color in the figure represents a sample, and the horizontal line in the figure represents the median gene expression in the sample. Figure S1C shows the relationship between exons and transcripts: The number of transcripts with the corresponding number of exons is counted. X-coordinate: Number of exons; y-coordinate: Number of transcripts with exons; Figure S1D relationship between genes and transcripts Bar chart: The number of genes with corresponding transcripts was counted. X-coordinate: Number of transcripts; Ordinate: The Number of genes corresponding to the number of transcripts

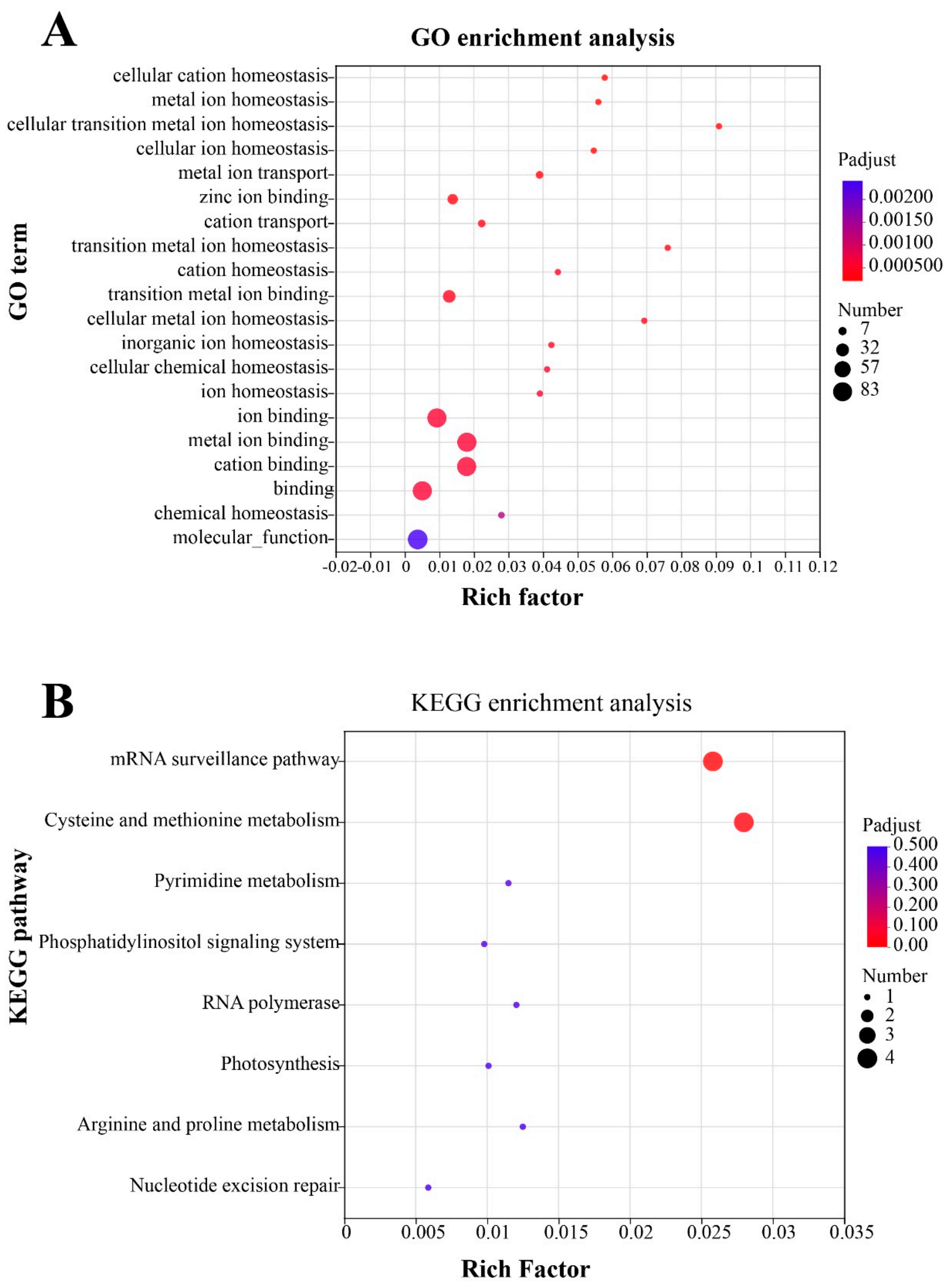

Figure S2. Functional enrichment analysis of cadmium-related genes; FIG. S2A shows the GO enriched bubble diagram. The vertical axis represents GO Term and the horizontal axis represents Rich factor. FIG. S2B shows the KEGG enriched bubble map, with pathway names on the vertical axis and Rich factor on the horizontal axis. The larger the Rich factor, the greater the degree of enrichment, the size of the dots indicated the number of genes in this GO Term, and the color of the dots corresponded to different Padjust ranges

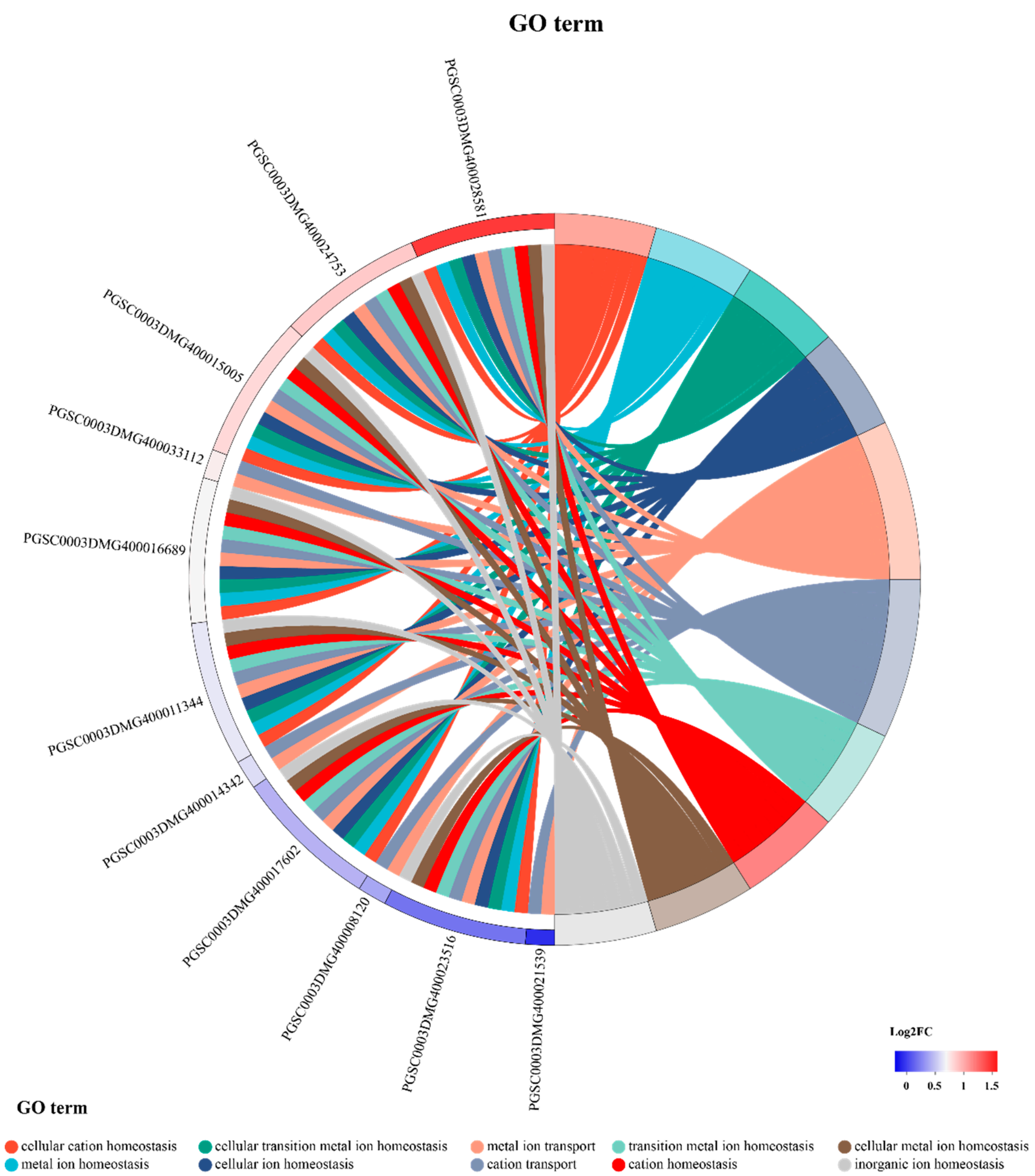

Figure S3. GO enriched chorography: Represents the significantly enriched GO term corresponding to differentially expressed genes/transcripts. On the left is the gene/transcript, which is arranged in the order of log2FC from largest to smallest. The larger log2FC is, the larger the differentially expressed multiples of up-regulated genes/transcripts, and the smaller logFC is, the larger the differentially expressed multiples of down-regulated genes/transcripts. The closer log2FC was to 0, the smaller the differential expression ratio of gene/transcript was. On the right is GO Term information on significantly enriched differential genes/transcripts.

```
             10        20        30        40        50        60
1    ATGAGCTCCCCTCCACAGCAAGAAAATACCTCTCCGGATTCCAAAGACGAAGAATCTCGT
1     M  S  S  P  P  Q  Q  E  N  T  S  P  D  S  K  D  E  E  S  R

             70        80        90       100       110       120
61   CACCTTCTTACCGCCCCGCTGCCACAGTCAACGTCGCCGTTAATTAACGGCGATGCTGAT
21    H  L  L  T  A  P  L  P  Q  S  T  S  P  L  I  N  G  D  A  D

            130       140       150       160       170       180
121  GACGGCGAAGAAGAATTTGCGTACGGATCTGGGGAGAAGATCCACGTCGTTGAATTCGAT
41    D  G  E  E  E  F  A  Y  G  S  G  E  K  I  H  V  V  E  F  D

            190       200       210       220       230       240
181  TCAGTACCGATCGACGGTGTTGATTATAGCACGGTGCCTCCATTCTCGTGGAAGAAGCTA
61    S  V  P  I  D  G  V  D  Y  S  T  V  P  P  F  S  W  K  K  L

            250       260       270       280       290       300
241  TGGCAGTTCACGGGTCCTGGATTCTTAATGAGTATAGCTTTTTGGATCCGGGGAATTTG
81    W  Q  F  T  G  P  G  F  L  M  S  I  A  F  L  D  P  G  N  L

            310       320       330       340       350       360
301  GAGGGGGATCTGCAAGCTGGGGCAATTGCGGGTTACTCGCTTCTATGGCTGTTGTTATGG
101   E  G  D  L  Q  A  G  A  I  A  G  Y  S  L  L  W  L  L  L  W

            370       380       390       400       410       420
361  GCCACTGTTATGGGTTTGATGATCCAGCTACTGTCGGCCAGAATTGGCGTTGTAACAGGC
121   A  T  V  M  G  L  M  I  Q  L  L  S  A  R  I  G  V  V  T  G

            430       440       450       460       470       480
421  CGGCACTTGGCGGAGCTTTGCCGGGAGGAGTATCCTAGATGGGCTGGGCTTCTACTGTGG
141   R  H  L  A  E  L  C  R  E  E  Y  P  R  W  A  G  L  L  L  W

            490       500       510       520       530       540
481  TTCATGGCTGAGGTGGCTCTGATTGGAGCCGATATTCAGGAGGTGATAGGGAGTGCCATT
161   F  M  A  E  V  A  L  I  G  A  D  I  Q  E  V  I  G  S  A  I

            550       560       570       580       590       600
541  GCAATTAAGATACTCAGTCGTGGGGTTTTACCACTCTGGGCTGGTGTCCTTATTACTGCT
181   A  I  K  I  L  S  R  G  V  L  P  L  W  A  G  V  L  I  T  A

            610       620       630       640       650       660
601  TCTGATTGCTTTCTTCTTTTGGTTCTTGAGAACTATGGTATAAGGAAGTTGGAAGCTGTG
201   S  D  C  F  L  L  L  V  L  E  N  Y  G  I  R  K  L  E  A  V

            670       680       690       700       710       720
661  TTTGCTGTTCTTATTTCGACTATGGCACTGTCCTTTGCTTGGATGTTTGGAGATGCAAAA
221   F  A  V  L  I  S  T  M  A  L  S  F  A  W  M  F  G  D  A  K

            730       740       750       760       770       780
721  CCAAATGGGAAGGAGCTTTTAGCAGGTCTCTTGATTCCAAAACTCAGTTCAAGGACAGTT
241   P  N  G  K  E  L  L  A  G  L  L  I  P  K  L  S  S  R  T  V

            790       800       810       820       830       840
781  CGGCAGGCTGTTGGAGTAGTTGGTTGTGTAATAATGCCTCACAATGTCTTCTTGCATTCA
261   R  Q  A  V  G  V  V  G  C  V  I  M  P  H  N  V  F  L  H  S

            970       980       990      1000      1010      1020
961  GTTACAACTGTCTTTGCCAAGGGATTTTATGGCAGTGAGCAAGCTGGTAGTTTAGGCCTT
321   V  T  T  V  F  A  K  G  F  Y  G  S  E  Q  A  G  S  L  G  L

           1030      1040      1050      1060      1070      1080
1021 GTAAATGCAGGGCAGTTTCTTCAGGACAAGTATGGTGGGGACTGTTCCCAATTCTCTAT
341   V  N  A  G  Q  F  L  Q  D  K  Y  G  G  G  L  F  P  I  L  Y

           1090      1100      1110      1120      1130      1140
1081 ATTTGGGGCATTGGGTTACTGGCAGCTGGGCAGAGTAGTACGATAACTGGTACTTATGCT
361   I  W  G  I  G  L  L  A  A  G  Q  S  S  T  I  T  G  T  Y  A

           1150      1160      1170      1180      1190      1200
1141 GGACAGTTTATTATGGGAGGTTTTCTAGATCTACGTTTGAAGAAATGGCTTAGGGCGCTG
381   G  Q  F  I  M  G  G  F  L  D  L  R  L  K  K  W  L  R  A  L

           1210      1220      1230      1240      1250      1260
1201 ATTACTCGAAGTTGTGCCATTGTGCCAACAATCATTGTCGCTCTGATTTTTAATAGATCT
401   I  T  R  S  C  A  I  V  P  T  I  I  V  A  L  I  F  N  R  S

           1270      1280      1290      1300      1310      1320
1261 GAATCATCACTCGACGTTTTGAATGAGTGGCTTAATGTGCTTCAGTCTATACAGATCCCT
421   E  S  S  L  D  V  L  N  E  W  L  N  V  L  Q  S  I  Q  I  P

           1330      1340      1350      1360      1370      1380
1321 TTTGCGCTTATTCCCCTTCTGACATTGGTGTCCAAGGAGGATATAATGGGTACTTTCAAA
441   F  A  L  I  P  L  L  T  L  V  S  K  E  D  I  M  G  T  F  K

           1390      1400      1410      1420      1430      1440
1381 ATTGGGCCTACTCTCGAGAGAGTTGCATGGACTATTGCTGTACTTGTGATGGTGATAAAC
461   I  G  P  T  L  E  R  V  A  W  T  I  A  V  L  V  M  V  I  N

           1450      1460      1470      1480      1490      1500
1441 GGCTATCTTTTGCTGGACTTCTTTGTCTCTGAGGTCAACGGACTGCTGTTTGCTTTTCTG
481   G  Y  L  L  L  D  F  F  V  S  E  V  N  G  L  L  F  A  F  L

           1510      1520      1530      1540      1550      1560
1501 GTCTGTGCTGGGACTGCAGGTTATATTGCCTTCATTTTATACCTTATTTCACATGGGGGT
501   V  C  A  G  T  A  G  Y  I  A  F  I  L  Y  L  I  S  H  G  G

           1570      1580      1590      1600      1610      1620
1561 GGCAATGTTGCCAATTGGTTCAACCTACTCCGCACAAAAGGATATAGCTATGCTGGTCAA
521   G  N  V  A  N  W  F  N  L  L  R  T  K  G  Y  S  Y  A  G  Q

1621 TGA
541   *
```

Figure S4. Amino acid sequence analysis of potato StNRAMP2 gene

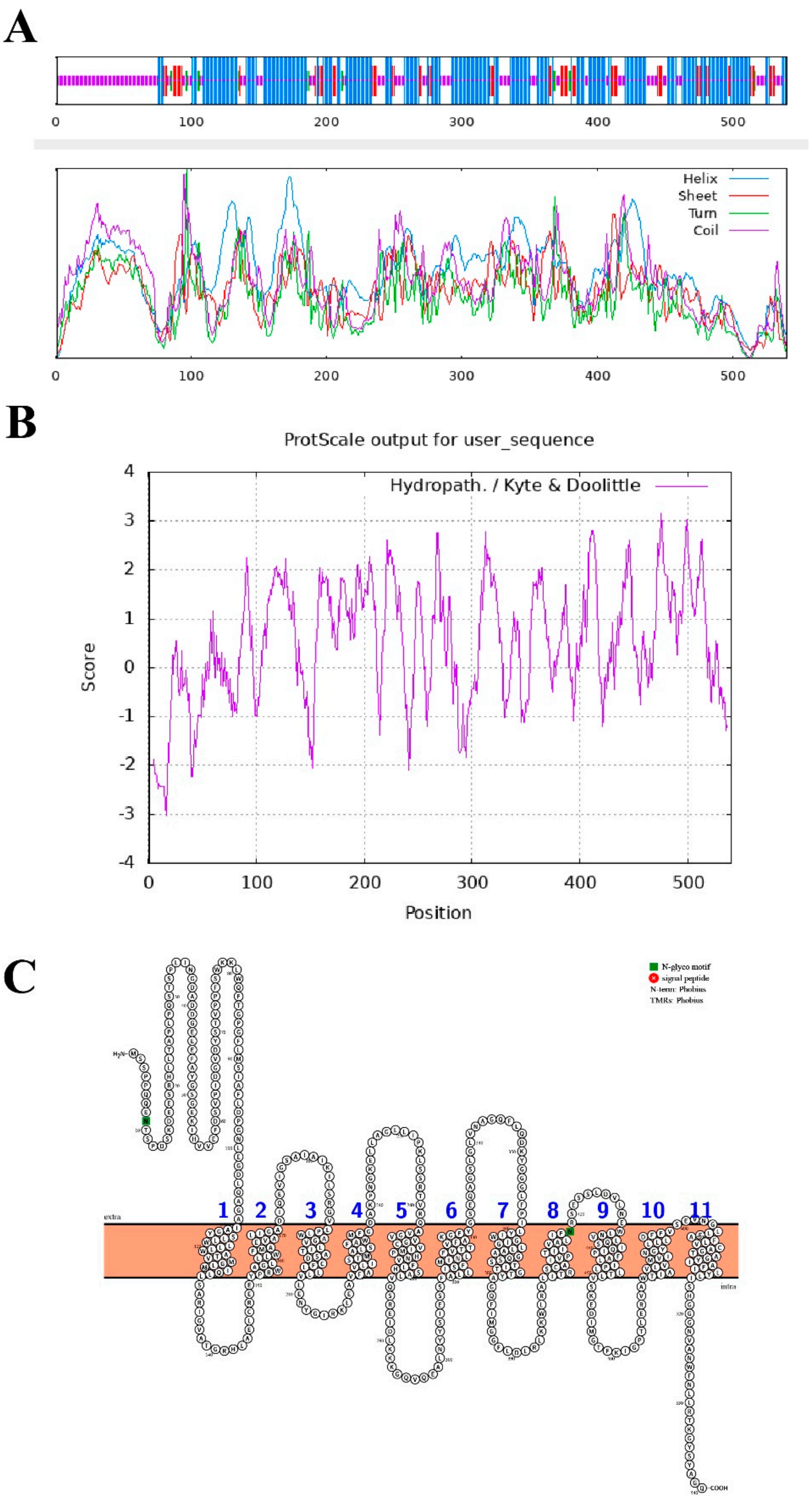

Figure S5. Qualitative *StNRAMP2*: Figure S5A is the second-level structure diagram of *StNRAMP2*; Figure S5B is the hydrophilic diagram of *StNRAMP2*: the peak value (positive value) of the figure represents the hydrophobic region, while the negative value of the "trough" region is the hydrophilic region; Figure S5C *StNRAMP2* transmembrane structure diagram

```
              10        20        30        40        50        60
1    GAATTCATGAGCTCCCCTCCACAGCAAGAAAATACCTCTCCGGATTCCAAAGACGAAGAA

              70        80        90       100       110       120
61   TCTCGTCACCTTCTTACCGCCCCGCTGCCACAGTCAACGTCGCCGTTAATTAACGGCGAT

             130       140       150       160       170       180
121  GCTGATGACGGCGAAGAAGAATTTGCGTACGGATCTGGGGAGAAGATCCACGTCGTTGAA

             190       200       210       220       230       240
181  TTCGATTCAGTACCGATCGACGGTGTTGATTATAGCACGGTGCCTCCATTCTCGTGGAAG

             250       260       270       280       290       300
241  AAGCTATGGCAGTTCACGGGCCCTGGATTCTTAATGAGTATAGCTTTTTTGGATCCGGGG

             310       320       330       340       350       360
301  AATTTGGAGGGGGATCTGCAAGCTGGGGCAATTGCGGGTTACTCGCTTCTATGGCTCTCG

361  AG
```

Figure S6. Sequencing results of StNRAMP2 gene silencing fragment in potato

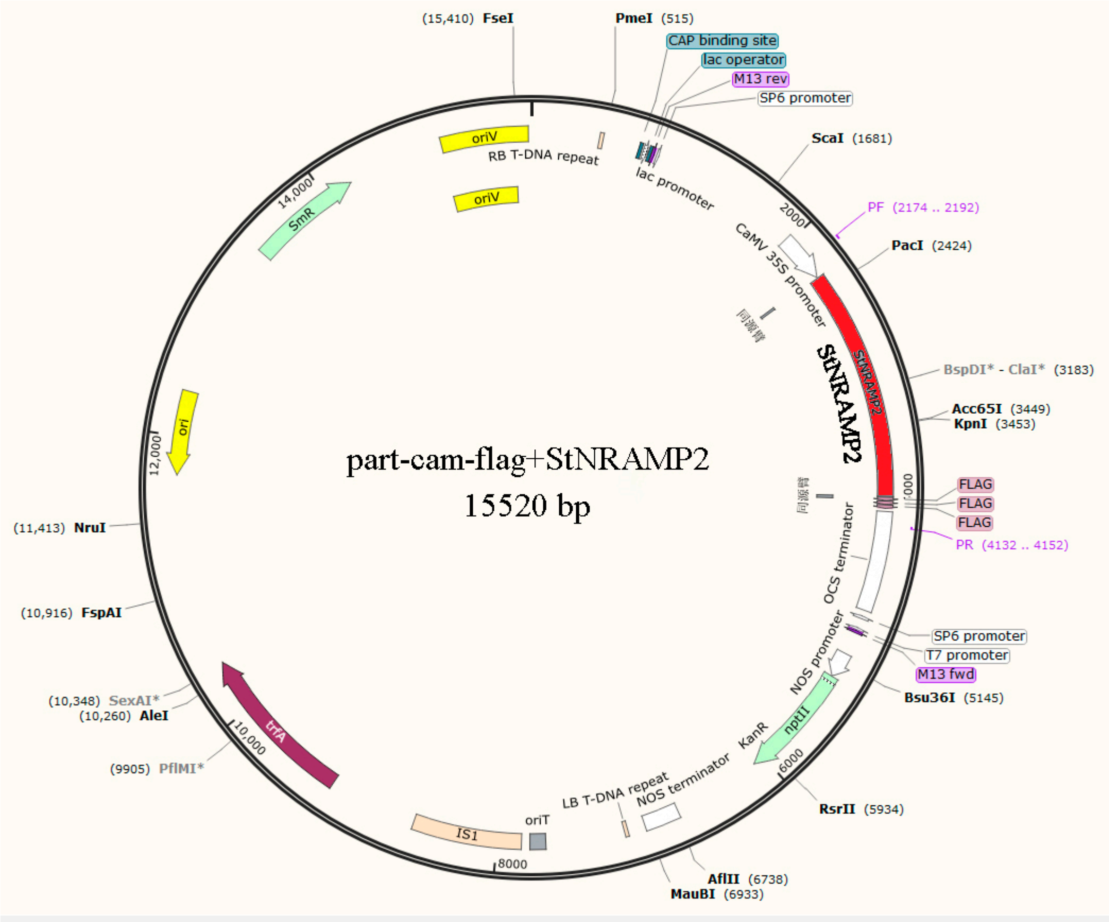

Figure S7. Map of StNRAMP2 recombinant plasmid

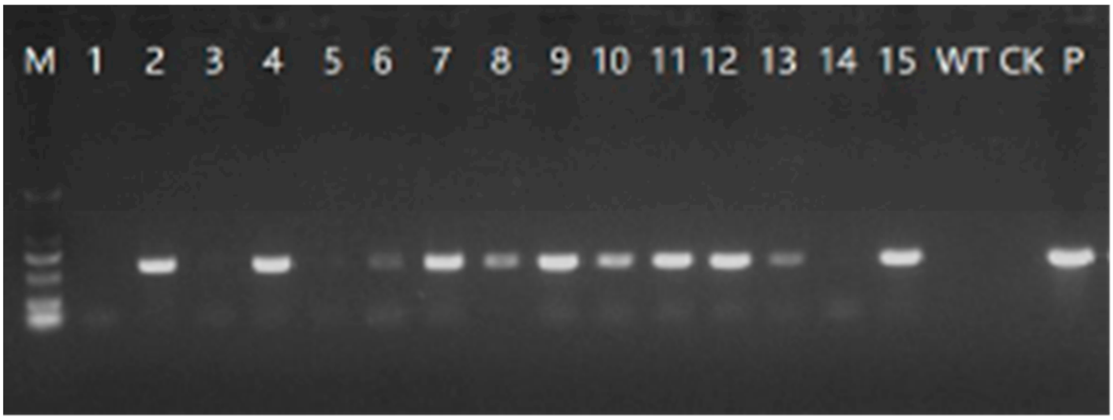

Figure S8. PCR results of StNRAMP2 transfection to tomato;M is DL2000 maker, 1-15 is the transgenic strain, WT is the negative control (wild type),CK is the blank control (water), P is the positive control (agrobacterium solution).
